# PCOS-GWAS Susceptibility Variants in *THADA, INSR, TOX3*, and *DENND1A* Are Associated With Metabolic Syndrome or Insulin Resistance in Women With PCOS

**DOI:** 10.3389/fendo.2020.00274

**Published:** 2020-04-30

**Authors:** Ye Tian, Jingyu Li, Shizhen Su, Yongzhi Cao, Zhao Wang, Shigang Zhao, Han Zhao

**Affiliations:** ^1^Center for Reproductive Medicine, Cheeloo College of Medicine, Shandong University, Jinan, China; ^2^Department of Gynecology and Obstetrics, Tianjin Medical University General Hospital, Tianjin, China; ^3^National Research Center for Assisted Reproductive Technology and Reproductive Genetics, Jinan, China; ^4^Key Laboratory of Reproductive Endocrinology of Ministry of Education, Shandong University, Jinan, China

**Keywords:** PCOS, MS, IR, GWAS, variants

## Abstract

Polycystic ovary syndrome is characterized by reproductive and metabolic disturbances throughout the female lifespan. Therefore, this study aimed to determine whether genome-wide association studies (GWAS)-identified risk variants for PCOS could confer risk of metabolic syndrome (MS) or insulin resistance (IR). Fifteen independent SNPs mapping to 11 GWAS loci genotyped in a total of 2,082 Han Chinese women independent of previous GWAS and phenotype-genotype correlations were assessed. The CC group for rs12478601 in *THADA* was associated with decreased rate of MS after adjustment for age (23.2 vs. 27%, *P* = 0.042, OR = 0.81). Using a dominant model, the GG+AG group for rs2059807 in *INSR* was associated with increased risk of MS after adjustment for age (26.8 vs. 22.5%, *P* = 0.023, OR = 1.27). The GG + GT group for rs4784165 in *TOX3* was found to be associated with an increased rate of IR after adjustment for age and BMI(53.3 vs. 48.5%, *P* = 0.027, OR = 1.27). The GG+AG group for rs2479106 in *DENND1A* was associated with a decreased rate of IR (48.3 vs. 53.6%, adjusted *P* = 0.039, OR = 0.80). After exclusion of PCOS cases with a family history of diabetes, hypertension, or dyslipidemia, the phenotype-genotype correlations between the genes *INSR* and *TOX3* and MS or IR were still significant (*P* < 0.05). Three SNPs (rs13429458 in *THADA*, rs10818854 in *DENND1A*, and rs2059807 in *INSR*) were significantly associated with IR; however, their association was not significant after adjustment for age and BMI. This genotype-phenotype study thus provides clues that *THADA, INSR, TOX3*, and *DENND1A* play a role in PCOS possibly through a metabolic disorder-related pathway.

## Introduction

Polycystic ovary syndrome (PCOS) is a common disorder estimated to affect 5–10% of women of reproductive age and 5.6% of Chinese women (1). PCOS is characterized by clustering of reproductive (oligo-/anovulation, menstrual irregularity, hirsutism, persistent acne, hyperandrogenism, infertility, and pregnancy complications) and metabolic disturbances (metabolic syndrome, type II diabetes mellitus, and cardiovascular disease) across the female lifespan (2).

Insulin resistance (IR) is universally accepted as a key pathophysiological feature of PCOS and metabolic syndrome (MS), regardless of obesity. IR is observed in approximately 50–70% of women with PCOS (3) and with more severity than that in women without PCOS. The common description of MS includes elevated waist circumference, increased triglycerides, decreased HDL-C, elevated blood pressure, and elevated fasting glucose according to the Adult Treatment Panel III criteria (4). Among women with PCOS, the prevalence of MS is reported to be 8.2% in Italy (5), 26.8% in China (1), and 43% in the USA (6). Meta-analysis of 16 studies, including both BMI and non-BMI matched studies, showed that women with PCOS had a 2-3 times higher risk of MS than women without PCOS (7).

Twin and familial studies revealed a strong evidence for the genetic basis of PCOS and IR. Increased prevalence of IR was observed in the first-degree relatives of women with PCOS (8). Candidate gene approaches identified associated genes for PCOS that are mainly involved in insulin-signaling and androgen-related pathways but lack consistent replication. Our group performed the first two genome-wide association studies (GWAS) in Han Chinese women and reported 11 susceptibility loci (15 risk variants) for PCOS (9, 10), including *INSR, THADA, LHCGR, FSHR, C9orf3, DENND1A, YAP1, RAB5B, HMGA2, TOX3*, and *SUMO1P1*. In this study, we conducted a phenotype-genotype correlation analysis to investigate the impact of these 15 SNPs on MS and IR in women with PCOS.

## Methods

### Subjects

The independent cohort consisted of 2,082 Han Chinese women with PCOS, recruited from the Center for Reproductive Medicine, Shandong University. PCOS was defined using the 2003 Rotterdam PCOS consensus criteria (11), which required two of the following conditions: oligo-ovulation or anovulation (OA, menstrual cycle length >35 days), clinical or biochemical hyperandrogenism (HA, Ferriman-Gallwey score ≥ 6 or circulating total testosterone ≥60 ng/dl) (9), and polycystic ovarian morphology (PCO, on ultrasound at least 12 follicles in one ovary and/or increased ovarian volume > 10 mL). Cases with congenital adrenal hyperplasia, androgen-secreting tumors, Cushing's syndrome, thyroid disease, and hyperprolactinemia were excluded. Among the 2,082 subjects, 157 had at least one parent with diabetes, 549 had at least one parent with hypertension, and 1 had at least one parent with dyslipidemia. Individuals who were taking medications such as oral contraceptives and metformin during the last 3 months were also excluded.

The study was approved by the Institutional Review Board of Center for Reproductive Medicine Shandong University and written informed consent was obtained from all participants.

### Clinical and Biochemical Measurement

All subjects were assessed for family history, blood pressure, height, weight, and waist circumference. The body mass index (BMI) was calculated as weight (kg)/height (m)^2^. Fasting blood samples were obtained during days 2–4 of the menstrual cycle to examine circulating serum levels of hormones such as total testosterone (T). Fasting plasma glucose levels were measured using the hexokinase method, and insulin levels were measured using the electrochemiluminescence method. Homeostasis model assessment (HOMA-IR) was calculated as fasting glucose (mmol/L) × fasting insulin (mIU/L) / 22.5, and 2.69 was used as the cut-off point for IR (12). HOMA-B was calculated as 20 × fasting insulin (mIU/L) / (fasting glucose (mmol/L) - 3.5) %. Serum triglycerides (TG) and high-density lipoprotein (HDL) were detected using enzymatic methods.

MS was defined by the 2005 Adult Treatment Panel III criteria (4), which required at least three of the following criteria: waist circumference ≥80 cm, serum TG ≥1.7 mmol/l, serum HDL cholesterol <1.3 mmol/l or the use of lipid lowering medication; blood pressure ≥130/85 mmHg or the use of anti-hypertensive medication, and fasting plasma glucose ≥5.6 mmol/l.

### SNP Genotyping

Genomic DNA was extracted from whole peripheral blood by a standard process using the QIAamp DNA mini kit (Qiagen). All 15 Chinese PCOS risk SNPs were genotyped using the Sequenom MassArray (Beijing, China).

### Statistical Analysis

Quantitative variables of clinical characteristics of the PCOS subjects were displayed as mean ± SD. Statistical analysis was performed using SPSS22.0 for Windows (SPSS, Inc., Chicago, IL, USA) and a *P* < 0.05 was considered statistically significant.

Genetic models were divided into additive (+/+ vs. +/– vs. –/–), dominant (+/+ plus +/– vs. –/–), and recessive (+/+ vs. +/– plus –/–). In the genotype-phenotype analysis, an appropriate genetic model was selected considering the small numbers in the homozygous minor allele groups. Categorical variables were compared using Pearson's chi-square (χ^2^) test and the results were adjusted for age and BMI using logistic regression. The odds ratios (ORs) were modeled to analyze the risk variants of MS and IR in PCOS and 95% confidence intervals (95% CIs) were presented.

## Results

### Clinical Features

The clinical characteristics of 2,082 PCOS subjects are displayed in Table 1. The average age of these women was 27.73 years and the average BMI was 24.67 kg/m^2^. The mean serum level of total testosterone was 47.22 ng/dl. The prevalence of MS in women with PCOS was 24.6% and the prevalence of IR was 50.7%. The frequency of the different components of MS was as follows: elevated waist circumference (38.1%), increased triglycerides (20.8%), decreased HDL-C (56.2%), elevated blood pressure (13.9%), and elevated fasting glucose (30.0%).

**Table 1:** Characteristics of 2082 PCOS cases.

**Variables**	**Value or number**
Age (years)	27.73 ± 3.93
BMI (kg/m^2^)	24.67 ± 5.16
Total testosterone (ng/dl)	47.22 ± 23.5
Waist circumference (cm)	86.21 ± 11.69
Triglycerides (mmol/l)	1.31 ± 0.85
High-density lipoprotein (mmol/l)	1.31 ± 0.34
Blood pressure (mmHg)	121.08 ± 13.59/79.25 ± 10.82
Fasting plasma glucose (mmol/l)	5.43 ± 0.92
HOMA-IR	3.31 ± 3.14
IR	50.7% (1,056/2,082)
MS	24.6% (512/2,082)

*BMI, body mass index; HOMA, homeostasis model assessment; IR, insulin resistance; MS, metabolic syndrome*.

### MS and IR Rate in the PCOS Subgroup With or Without Hyperandrogenism (HA)

Except for 52 cases with HA status unavailable, 820 cases presented with HA (including 27 HA + OA, 36 HA + PCO and 757 HA + OA + PCO), whereas 1,210 cases presented without HA (including 1,210 OA + PCO). In PCOS women with or without the HA subgroup, the rates of MS were similar after adjustment for age (24.9 vs. 24.5%, *P* = 0.703 OR = 1.04, Table 2). The rate of IR in the HA group was higher than that in the non-HA group, but did not reach significant levels (55 vs. 48.8%, *P* = 0.278 OR = 1.13, Table 2).

**Table 2:** Metabolic syndrome and insulin resistance in HA and non-HA women with PCOS.

		**Metabolic syndrome**				**Insulin resistance**			
	**N**	**MS**	**MS/Non-MS**	***P*^a^**	**OR^a^**	**IR**	**IR/Non-IR**	***P*^b^**	**OR^b^**
HA subgroup	820	24.9%	204/616	0.703	1.04 (0.85–1.28)	55.0%	449/368	0.278	1.13 (0.91–1.40)
Non-HA subgroup	1,210	24.5%	297/913			48.8%	584/612		

a *adjusted P by age*,

b *adjusted P by age and BMI*.

*HA, hyperandrogenism; IR, insulin resistance; MS, metabolic syndrome; OR: odds ratio*.

### Genotype-Phenotype Analysis of 15 SNPs and MS

The allele frequencies of 15 SNPs are shown in Supplementary Table 1. For phenotypic and genotypic assessment, an appropriate dominant or recessive genetic model was selected considering the small numbers in the homozygous minor allele groups (Table 3), and the additive model was also performed in Supplementary Table 2. Using a recessive model, the CC group for rs12478601 in *THADA* was associated with decreased rate of MS (23.2 vs 27%, *P* = 0.051), and the association was significant after adjustment for age (*P* = 0.042, OR = 0.81). Using a dominant model, the rate of MS was significantly higher in the GG + AG group for rs2059807 in *INSR* than in the AA group (26.8 vs. 22.5%, *P* = 0.023, OR = 1.26, Table 3), even after adjustment for age (adjusted *P* = 0.023, OR = 1.27), indicating that the risk genotype of rs2059807 was robustly associated with MS in PCOS cases. After exclusion of PCOS cases with a family history of diabetes or hypertension or dyslipidemia, the phenotype-genotype correlations between the gene *INSR* and MS were still significant, whereas the correlation between *THADA* and MS was not significant (rs2059807, age-adjusted *P* = 0.005, OR = 1.43; rs12478601, age-adjusted *P* = 0.067, OR = 0.79). No association between the genotype of rs12478601, rs2059807 and waist circumference, blood pressure, TG, HDL-C were identified (Supplementary Table 3). The rate of MS in the AA group for rs2479106 in *DENND1A*, and the GG+GT group for rs4784165 in *TOX3*, were all slightly higher than that in another genotype group using dominant/recessive model (26.4 vs. 22.9%, *P* = 0.066; 26.3 vs. 22.7%, *P* = 0.068, Table 3). For rs4784165, the results were similar using additive model (Supplementary Table 2).

**Table 3:** Association of MS and the genotypes of 15 SNPs using dominant/recessive model.

**SNP**	**Reported gene**	**Genotype**	**MS**	***P***	***P*^a^**	**OR^a^**
rs13429458	*THADA*	AA/AC+CC	24.2%/26.3%	0.324	0.339	0.90 (0.72–1.12)
rs13405728	*LHCGR*	AA/AG+GG	23.6%/27.0%	0.088	0.082	0.83 (0.67–1.02)
rs12478601	*THADA*	CC/TC+TT	23.2%/27.0%	0.051	0.042	0.81 (0.66–0.99)
rs10818854	*DENND1A*	AA+AG/GG	22.2%/25.4%	0.177	0.148	0.83 (0.64–1.07)
rs2479106	*DENND1A*	GG+AG/AA	22.9%/26.4%	0.066	0.063	0.82 (0.67–1.01)
rs2268361	*FSHR*	CC/TC+TT	25.1%/24.7%	0.839	0.872	1.02 (0.81–1.28)
rs2349415	*FSHR*	TT+TC/CC	23.9%/25.4%	0.462	0.369	0.91 (0.73–1.12)
rs4385527	*C9orf3*	GG /AG+AA	24.7%/24.8%	0.955	0.978	0.99 (0.80–1.24)
rs3802457	*C9orf3*	GG /AG+AA	24.2%/28.0%	0.171	0.140	0.81 (0.61–1.07)
rs1894116	*YAP1*	GG+AG/AA	23.9%/25.4%	0.419	0.382	0.91 (0.74–1.12)
rs705702	*RAB5B*	GG+AG/AA	25.3%/24.3%	0.613	0.549	1.06 (0.87–1.30)
rs2272046	*HMGA2*	AA/AC+CC	24.3%/27.7%	0.214	0.180	0.83 (0.62–1.09)
rs4784165	*TOX3*	GG+GT/ TT	26.3%/22.7%	0.068	0.084	1.20 (0.98–1.48)
rs2059807	*INSR*	GG+AG/AA	26.8%/22.5%	0.023	0.023	1.27 (1.03–1.55)
rs6022786	*SUMO1P1*	AA+AG/GG	25.1%/24.2%	0.643	0.677	1.05 (0.85–1.28)

a *adjusted P by age*.

*MS, metabolic syndrome; OR: odds ratio*.

### Genotype-Phenotype Analysis of 15 SNPs and IR

The association of HOMA-IR and the genotypes of 15 SNPs are shown in Table 4 and Supplementary Table 4. The GG + GT group for rs4784165 in *TOX3* was associated with an increased rate of IR (53.3 vs. 48.5%, *P* = 0.027, OR = 1.27, Table 4) using a dominant model after adjustment for age and BMI. The GG+AG group for rs2479106 in *DENND1A* was associated with a decreased rate of IR using a dominant model (48.3 vs. 53.6%, age- and BMI-adjusted *P* = 0.036, OR = 0.80, Table 4). Three SNPs (rs13429458 in *THADA*, rs10818854 in *DENND1A*, and rs2059807 in *INSR*) were significantly associated with IR; however, the associations were not significant after adjustment for age and BMI (Table 4). Using additive model, only rs13429458 was significantly associated with IR after adjustment for age and BMI (Supplementary Table 4). After exclusion of subjects with a family history of diabetes, the phenotype-genotype correlations between gene *TOX3* and IR were still significant, whereas the correlation between *DENND1A* and IR was not significant (rs4784165, age- and BMI-adjusted *P* = 0.031, OR = 1.28; rs2479106, age- and BMI-adjusted *P* = 0.053, OR = 0.81).

**Table 4:** Association of HOMA-IR and the genotypes of 15 SNPs using dominant/recessive model.

**SNP**	**Reported gene**	**Genotype**	**IR**	***P***	***P*^a^**	**OR^a^**
rs13429458	*THADA*	AA/AC+CC	49.7%/55.6%	0.019	0.056	0.8 (0.63–1.01)
rs13405728	*LHCGR*	AA/AG+GG	50.1%/53.5%	0.151	0.649	0.95 (0.76–1.19)
rs12478601	*THADA*	CC/TC+TT	49.9%/53.0%	0.165	0.767	0.97 (0.78–1.2)
rs10818854	*DENND1A*	AA+AG/GG	45.9%/52.6%	0.015	0.220	0.85(0.66-1.1)
rs2479106	*DENND1A*	GG+AG/AA	48.3%/53.6%	0.017	0.039	0.8 (0.65–0.99)
rs2268361	*FSHR*	CC/TC+TT	51.6%/51.2%	0.089	0.568	0.93(0.74-1.18)
rs2349415	*FSHR*	TT+TC/CC	50.4%/51.8%	0.557	0.485	0.92 (0.74–1.15)
rs4385527	*C9orf3*	GG/AG+AA	51.3%/51.0%	0.882	0.468	0.92 (0.73–1.16)
rs3802457	*C9orf3*	GG/AG+AA	51.0%/52.1%	0.722	0.899	0.98 (0.73–1.32)
rs1894116	*YAP1*	GG+AG/AA	51.3%/51.1%	0.916	0.687	1.04 (0.84–1.29)
rs705702	*RAB5B*	GG+AG/AA	52.9%/49.8%	0.169	0.727	1.04 (0.84–1.28)
rs2272046	*HMGA2*	AA/AC+CC	51.3%/49.8%	0.634	0.526	1.1 (0.81–1.49)
rs4784165	*TOX3*	GG+GT/TT	53.3%/48.5%	0.034	0.027	1.27 (1.03–1.58)
rs2059807	*INSR*	GG+AG/AA	53.4%/48.7%	0.035	0.099	1.19 (0.97–1.48)
rs6022786	*SUMO1P1*	AA+AG/GG	52.2%/49.7%	0.273	0.591	1.06 (0.86–1.31)

a *adjusted P by age and BMI*.

*IR, insulin resistance; OR: odds ratio*.

The genotype-phenotype analysis was also performed for 15 SNPs and HOMA-B, fasting insulin, and TG/HDL-C (Supplementary Table 5). Rs10818854 in *DENND1A* and rs2059807 in *INSR* were associated with HOMA-B (*P* < 0.05). Rs4784165 in *TOX3* was associated with TG/HDL-C (*P* < 0.05).

## Discussion

Previously our group performed the first two GWAS on women with PCOS and the results were replicated in a large number of cohorts with different ethnicities (13–19). The relationship between important clinical features of PCOS and these GWAS-identified variations have been examined in several studies. To the best of our knowledge, this is the first report on the relation of MS and PCOS-GWAS risk variations. In the present study, we performed genotype-phenotype assessment and found that PCOS susceptibility variants in *THADA* and *INSR*, identified in previous GWAS, confers risks for MS in women with PCOS, and that variants in *DENND1A* and *TOX3* were associated with IR.

PCOS is associated with increased risk of metabolic dysfunction including MS and IR. Individuals with MS are at increased risk for serious complications in type 2 diabetes (T2D) and cardiovascular disease (CVD) (20–22). In this study, the rate of MS in women with PCOS was 24.6%, which is lower than that observed in women from the USA (43%) and higher than that in women from Italy (8.2%). The rate of MS did not differ in PCOS subgroups with or without HA. Consistent with our data, a large-scale epidemiological study in reproductive-aged Han Chinese women showed that the prevalence of MS in women with PCOS was 26.8% and did not differ among four different subtypes of PCOS based on the Rotterdam criteria (1).

*THADA* (thyroid adenoma associated), was initially identified in thyroid adenomas. *THADA* was identified as a risk locus for T2D by GWAS (23) and was replicated in Indian sib pairs (24). Gene variant in *THADA* was associated with pancreatic b-cell response (25). However, the association between *THADA* and T2D were not replicated by some studies (26, 27). In this study, rs12478601 and rs13429458 in *THADA* were not significantly associated with IR after adjustment for age and BMI. The relation between *THADA* and PCOS was firstly identified in a GWAS by our group (9) and replicated by other cohorts (13, 16). Further, its relation to PCOS was confirmed by family-based study using transmission disequilibrium test (TDT) (28). In the cross-ethnic meta-analysis of the Chinese, US and Dutch patients, the association between *THADA* and PCOS was confirmed across populations (29). In this study, SNP rs12478601 in *THADA* was associated with MS in PCOS, indicating *THADA* may play an important role in metabolism. The decreased risk of MS associated with CC genotype provide speculation that *THADA* may affect the pathogenesis of MS-PCOS and non-MS-PCOS through an independent pathway. In consistent with our study, variation in *THADA* was strongly associated with total cholesterol and low-density lipoprotein in Mexican Americans (30). *THADA* was predicted to involve in adipogenesis (31). In adipose tissues of women with PCOS carried rs12478601-C, the response to metformin with lower basal glucose was more significant (31). *THADA* knockout *Drosophila* were obese and produce less energy than controls. THADA bind the sarco/ER Ca^2+^ ATPase (SERCA) and regulated metabolism through calcium signaling (32).

The *INSR* gene is composed of 22 exons and encodes the insulin receptor, a heterotetrameric glycoprotein belonging to the tyrosine kinase receptor family. Knockout of *Insr* gene in mice causes extreme insulin resistance (33). Altered INSR expression causes IR and diabetes in humans and mice (34, 35). IR in individuals with PCOS is associated with decreased tyrosine autophosphorylation of the insulin receptor considering that the number and affinity of INSR is not altered (36). Single nucleotide polymorphisms in the *INSR* gene may introduce changes in insulin receptor function and may be associated with PCOS (37). A number of genetic association studies have been conducted in PCOS case-control cohorts, most of which have focused on a silent C/T variation in codon His 1,058; however, their results were inconsistent in different populations (38). The very important validation of the *INSR* gene as a PCOS risk gene was obtained in a large and well-designed case-control GWAS conducted by our group (10). The SNP rs2059807 in the *INSR* gene was discovered as an association signal and allele G was observed to be the risk allele for PCOS. In the present study, the risk genotype group GG+AG presented a higher rate of MS and IR than the AA group. Elouej et al. examined the association of rs2059807 and metabolic syndrome in 356 samples (male and female) from the Tunisian population and found that rs2059807 was not associated with a risk of MS (39). These conflicting results may arise due to the different criteria used to define MS and different genders.

*TOX3* gene encodes a nuclear protein belonging to the high-mobility group (HMG) box family. TOX3 is a calcium-dependent neuronal transcription factor and is involved in protecting neuronal cells from cell death. TOX3 can mediate cytoprotective transcription from different promoters (either BCL-2 promoter or complement C3 promoter), depending on the presence of different components of the transcriptionally active complex (either phosphorylated CREB or CITED1). *TOX3* was recently reported as a breast cancer susceptibility gene by large-scale GWAS (40, 41). TOX3 may play dual roles in cancer initiation and progression considering the decreased expression of TOX3 mRNA in breast cancers and increased expression of TOX3 mRNA in metastatic breast cancer (42, 43). *TOX3* was first identified as a PCOS susceptibility gene by our previous GWAS and allele G at rs4784165 was found as the risk allele for PCOS. Recently, the association between *TOX3* gene and PCOS was confirmed in another GWAS conducted by Chen Li et al. (44). However, the functional mechanism of TOX3 in PCOS pathogenesis and traits are unclear. Recently an abstract reported that *Tox3* knockout rats present an obesity phenotype, male and female sterility, and a behavioral phenotype (increased anxiety). In the present study, the risk genotype group, GG+GT, presented a higher rate of IR than the TT group in women with PCOS, indicating that *TOX3* may contribute to PCOS through a metabolic disorder-related pathway. Pau et al. conducted RNA sequencing for subcutaneous adipose tissue in individuals with PCOS, and found that TOX3 may be involved in inflammation (31). Sequencing of exons and the exon-intron boundary regions of the *TOX3* gene did not reveal any pathogenic mutations in 200 women with PCOS (45). The expression of TOX3 was lower in the serum and granulosa cells of PCOS subjects compared with that in the control group (46). The putative role of TOX3 in the etiology of PCOS thus needs further explanation.

*DENND1A* gene encodes the protein DENN/MADD domain containing 1A, which is involved in endosomal membrane trafficking. DENND1A is widely expressed and its relation to PCOS was originally detected in a GWAS by our group. A second GWAS, also conducted by our group, further confirmed that *DENND1A* gene is a PCOS-susceptibility gene and that allele G at rs2479106 is a risk allele for PCOS. *DENND1A* has been validated in European cohorts of women with PCOS, but rs2479106 was not shown to be a strongly associated SNP (13, 14). The relationship between quantitative traits and risk SNP rs2479106 has been further examined to better understand its potential function. In the present study, the risk GG+AG genotype group for rs2479106 was associated with a decreased rate of IR. It is speculated that *DENND1A* may affect the development of IR-PCOS and non-IR PCOS through an independent pathway. Allele G at rs2479106 was associated with increased waist-to-hip ratio(WHR), Chol/HDL, and LDL levels in a European cohort (47), whereas it was associated with increased 2-h insulin levels during oral glucose tolerance tests (OGTT) in a Chinese cohort (48). Besides, allele G at rs2479106 was found to confer risk of endometrioid adenocarcinoma (49), in which insulin resistance may play an important role. Related genetic studies have provided novel biological insights for DENND1A and two principal transcripts, DENND1A variant 1 (DENND1A.V1) and DENND1A variant 2 (DENND1A.V2) have been detected. The expression levels of DENND1A.V2 protein and mRNA are increased in PCOS theca cells. Knockdown of DENND1A.V2 in PCOS theca cells resulted in decreased androgen biosynthesis. Consistently, overexpression of DENND1A.V2 in normal theca cells resulted in a PCOS phenotype including increased androgen biosynthesis (50). DENND1A.V2 thus plays a key role in hyperandrogenemia, indicating a possible biological relationship with IR.

There were some limitations in this study. First, the prevalence of MS is known to increase with age. The participants in this study were young. We thus suggest that future studies should examine the long-term risks of MS and IR in different genotype groups of women at advanced ages. Second, no multiple testing correction has been applied; hence all associations are nominally significant at 5%.

The genotype-phenotype analysis of PCOS GWAS loci is intended to shed light on the mechanism by which the risk SNPs may influence the pathogenesis of PCOS status. This genotype-phenotype study provides clues that *THADA, INSR, TOX3*, and *DENND1A* play important role in the etiology of PCOS, possibly through insulin resistance and metabolic disorder related pathways. The four variants, rs12478601, rs2059807, rs4784165, and rs2479106 are located in the intron regions of *THADA, INSR, TOX3*, and *DENND1A* genes, respectively; thus, it is unclear how these SNPs might affect the gene expression or function to influence the PCOS phenotype. These SNPs may associate with other functional causal variants in the region. A functional study of these three genes and their causal variants is thus needed to understand how they contribute to the biology of PCOS.

## Conclusion

PCOS susceptibility variants in *THADA* and *INSR* are associated with metabolic syndrome and variants in *TOX3* and *DENND1A* are associated with insulin resistance. This is the first report on the relation of metabolic syndrome (defined by 2005 Adult Treatment Panel III criteria) and PCOS-GWAS risk variations. It suggests that *THADA, INSR, TOX3*, and *DENND1A* might play a role in PCOS through a metabolic disorder related pathway.

## Data Availability Statement

All datasets generated for this study are included in the article/Supplementary Material.

## Ethics Statement

The studies involving human participants were reviewed and approved by Institutional Review Board of Center for Reproductive Medicine, Shandong University. The patients/participants provided their written informed consent to participate in this study.

## Author Contributions

HZ and SZ designed and supported the study and revised the article. YC and ZW collected all the clinical data and blood samples. YT, JL, and SS performed the experiments. YT analyzed the data and drafted the manuscript. All authors gave their final approval for the version to be published.

## Conflict of Interest

The authors declare that the research was conducted in the absence of any commercial or financial relationships that could be construed as a potential conflict of interest.
