# Supplementary material for: PCOS-GWAS Susceptibility Variants in THADA, INSR, TOX3, and DENND1A Are Associated With Metabolic Syndrome or Insulin Resistance in Women With PCOS
Source: Front Endocrinol (Lausanne). 2020 Apr 30;11:274. doi: 10.3389/fendo.2020.00274 (PMC7203965; doi:10.3389/fendo.2020.00274)
Supplement: Supplementary file 1 [file Data_Sheet_1.docx]

| **SNP** | **gene** | **MAF** | | ***P*** | **MAF** | | ***P*** |
| --- | --- | --- | --- | --- | --- | --- | --- |
|  |  | **MS** | **Non-MS** |  | **IR** | **Non-IR** |  |
| rs13429458 | *THADA* | 0.157 | 0.144 | 0.336 | 0.161 | 0.134 | 0.019 |
| rs13405728 | *LHCGR* | 0.206 | 0.179 | 0.060 | 0.195 | 0.177 | 0.150 |
| rs12478601 | *THADA* | 0.261 | 0.232 | 0.058 | 0.248 | 0.230 | 0.183 |
| rs10818854 | *DENND1A* | 0.097 | 0.113 | 0.144 | 0.097 | 0.121 | 0.014 |
| rs2479106 | *DENND1A* | 0.248 | 0.266 | 0.272 | 0.247 | 0.276 | 0.039 |
| rs2268361 | *FSHR* | 0.489 | 0.487 | 0.920 | 0.496 | 0.481 | 0.357 |
| rs2349415 | *FSHR* | 0.182 | 0.198 | 0.252 | 0.192 | 0.195 | 0.826 |
| rs4385527 | *C9orf3* | 0.168 | 0.162 | 0.685 | 0.164 | 0.164 | 0.946 |
| rs3802457 | *C9orf3* | 0.080 | 0.067 | 0.157 | 0.072 | 0.068 | 0.606 |
| rs1894116 | *YAP1* | 0.218 | 0.243 | 0.114 | 0.234 | 0.241 | 0.597 |
| rs705702 | *RAB5B* | 0.300 | 0.292 | 0.646 | 0.304 | 0.287 | 0.235 |
| rs2272046 | *HMGA2* | 0.082 | 0.070 | 0.225 | 0.071 | 0.075 | 0.599 |
| rs4784165 | *TOX3* | 0.385 | 0.353 | 0.067 | 0.369 | 0.351 | 0.214 |
| rs2059807 | *INSR* | 0.326 | 0.299 | 0.107 | 0.320 | 0.292 | 0.047 |
| rs6022786 | *SUMO1P1* | 0.349 | 0.349 | 0.990 | 0.356 | 0.342 | 0.374 |

**Supplementary Table 1: Comparison of allele frequencies in MS/non-MS and IR/non-IR cases**

MS, metabolic syndrome; IR, insulin resistance.

**Supplementary Table 2:** **Association of MS and the genotypes of 15 SNPs using additive model**

| **SNP** | **Reported gene** | **genotype** | ***P*** | **OR** | **95% CI** |
| --- | --- | --- | --- | --- | --- |
| rs13429458 | *THADA* | AA / AC / CC | 0.332 | 0.91 | 0.74-1.11 |
| rs13405728 | *LHCGR* | AA / AG / GG | 0.061 | 0.84 | 0.71-1.01 |
| rs12478601 | *THADA* | CC / TC / TT | 0.056 | 0.85 | 0.72-1 |
| rs10818854 | *DENND1A* | AA / AG / GG | 0.148 | 0.84 | 0.67-1.06 |
| rs2479106 | *DENND1A* | GG / AG / AA | 0.275 | 0.91 | 0.78-1.07 |
| rs2268361 | *FSHR* | CC / TC / TT | 0.920 | 0.99 | 0.86-1.15 |
| rs2349415 | *FSHR* | TT / TC / CC | 0.251 | 0.9 | 0.75-1.08 |
| rs4385527 | *C9orf3* | GG /AG / AA | 0.685 | 0.96 | 0.79-1.16 |
| rs3802457 | *C9orf3* | GG /AG / AA | 0.149 | 0.82 | 0.62-1.07 |
| rs1894116 | *YAP1* | GG / AG / AA | 0.113 | 0.87 | 0.73-1.03 |
| rs705702 | *RAB5B* | GG / AG / AA | 0.639 | 1.04 | 0.89-1.22 |
| rs2272046 | *HMGA2* | AA / AC / CC | 0.226 | 0.85 | 0.65-1.11 |
| rs4784165 | *TOX3* | GG / GT/ TT | 0.068 | 1.15 | 0.99-1.33 |
| rs2059807 | *INSR* | GG / AG / AA | 0.099 | 1.14 | 0.98-1.33 |
| rs6022786 | *SUMO1P1* | AA / AG / GG | 0.990 | 1 | 0.86-1.16 |

MS, metabolic syndrome; OR: odds ratio.

**Supplementary Table 3: Association of MS parameters and the genotypes of 2 SNPs**

| **SNP** | **rs2059807** | | ***P*** | **rs12478601** | | ***P*** |
| --- | --- | --- | --- | --- | --- | --- |
|  | **GG+AG** | **AA** |  | **CC** | **TC+TT** |  |
| WC (cm) | 86.61±11.68 | 85.83±11.78 | 0.137 | 86.81±12.13 | 85.87±11.4 | 0.075 |
| SBP (mmHg) | 121.44±13.74 | 120.72±13.54 | 0.239 | 121.39±13.51 | 120.93±13.69 | 0.446 |
| DBP (mmHg) | 79.52±10.89 | 78.95±10.85 | 0.244 | 79.77±10.81 | 78.93±10.84 | 0.084 |
| fasting glucose(mmol/l) | 5.42±0.98 | 5.45±0.86 | 0.411 | 5.45±0.86 | 5.43±0.97 | 0.666 |
| TG (mmol/l) | 1.31±0.84 | 1.3±0.84 | 0.845 | 1.32±0.88 | 1.3±0.83 | 0.505 |
| HDL-C (mmol/l) | 1.3±0.33 | 1.31±0.36 | 0.651 | 1.31±0.36 | 1.3±0.33 | 0.462 |

WC: waist circumference; SBP: systolic blood pressure; DBP: diastolic blood pressure; TG: triglycerides; HDL-C: high-density lipoprotein cholesterol.

**Supplementary Table 4: Association of HOMA-IR and the genotypes of 15 SNPs using additive model**

| **SNP** | **Reported gene** | **genotype** | ***P*** | ***P* ^a^** | **OR ^a^** |
| --- | --- | --- | --- | --- | --- |
| **rs13429458** | ***THADA*** | **AA / AC / CC** | **0.018** | **0.036** | **0.80** |
| rs13405728 | *LHCGR* | AA / AG / GG | 0.150 | 0.878 | 0.99 |
| rs12478601 | *THADA* | CC / TC / TT | 0.180 | 0.745 | 0.97 |
| rs10818854 | *DENND1A* | AA / AG / GG | 0.015 | 0.251 | 0.87 |
| rs2479106 | *DENND1A* | GG / AG / AA | 0.040 | 0.054 | 0.85 |
| rs2268361 | *FSHR* | CC / TC / TT | 0.355 | 0.102 | 0.88 |
| rs2349415 | *FSHR* | TT / TC / CC | 0.826 | 0.691 | 0.96 |
| rs4385527 | *C9orf3* | GG /AG / AA | 0.946 | 0.506 | 0.93 |
| rs3802457 | *C9orf3* | GG /AG / AA | 0.599 | 0.799 | 0.96 |
| rs1894116 | *YAP1* | GG / AG / AA | 0.596 | 0.915 | 0.99 |
| rs705702 | *RAB5B* | GG / AG / AA | 0.225 | 0.860 | 1.02 |
| rs2272046 | *HMGA2* | AA / AC / CC | 0.599 | 0.347 | 1.15 |
| rs4784165 | *TOX3* | GG / GT/ TT | 0.216 | 0.108 | 1.14 |
| rs2059807 | *INSR* | GG / AG / AA | 0.043 | 0.093 | 1.15 |
| rs6022786 | *SUMO1P1* | AA / AG / GG | 0.369 | 0.681 | 1.03 |

**^a^** adjusted *P* by age and BMI

IR, insulin resistance; OR: odds ratio.

**Supplementary Table 5: Association of genotypes of 15 SNPs and HOMA-B, fasting insulin, TG/HDL-C**

| **SNP** | **Group1/2** | **HOMA-B** | | ***P*** | **fasting insulin** | | ***P*** | **TG/HDL-C** | | ***P*** |
| --- | --- | --- | --- | --- | --- | --- | --- | --- | --- | --- |
|  |  | **Group1** | **Group2** |  | **Group1** | **Group2** |  | **Group1** | **Group2** |  |
| rs13429458 | AA / AC+CC | 151.08±125.43 | 158.67±153.89 | 0.252 | 13.27±8.95 | 14.17±10.88 | 0.056 | 1.09±0.84 | 1.13±0.93 | 0.438 |
| rs13405728 | AA / AG+GG | 150.3±122.95 | 159.18±153.29 | 0.158 | 13.35±10.02 | 13.88±8.5 | 0.229 | **1.07±0.83** | **1.16±0.91** | **0.028** |
| rs12478601 | CC / TC+TT | 150.84±122.64 | 154.39±136.8 | 0.539 | 13.2±8.97 | 13.97±10.22 | 0.070 | 1.1±0.85 | 1.11±0.9 | 0.746 |
| rs10818854 | AA+AG / GG | **138.94±96.61** | **156.61±141.46** | **0.016** | 5.43±1.04 | 5.44±0.89 | 0.954 | 1.05±0.87 | 1.11±0.86 | 0.191 |
| rs2479106 | GG+AG / AA | 151.61±137.64 | 152.69±122.25 | 0.851 | 13.18±9.28 | 13.8±9.74 | 0.142 | 1.09±0.9 | 1.12±0.84 | 0.418 |
| rs2268361 | CC / TC+TT | 150.42±121.21 | 154.64±138.41 | 0.533 | 13.57±9.5 | 13.52±9.56 | 0.914 | 1.11±0.96 | 1.1±0.83 | 0.867 |
| rs2349415 | TT+TC / CC | 146.89±137.21 | 156.86±132.13 | 0.109 | 13.02±8.38 | 13.8±10.09 | 0.079 | 1.1±0.86 | 1.11±0.87 | 0.790 |
| rs4385527 | GG /AG+AA | 156.1±142.01 | 147.02±113.45 | 0.161 | 13.64±9.97 | 13.25±8.45 | 0.400 | 1.09±0.81 | 1.13±0.97 | 0.426 |
| rs3802457 | GG /AG+AA | 152.24±133.93 | 158.95±132.7 | 0.434 | 13.46±9.57 | 13.79±9.18 | 0.585 | 1.08±0.83 | 1.21±1.08 | 0.060 |
| rs1894116 | GG+AG / AA | 153.89±130.47 | 152.92±136.78 | 0.872 | 13.52±9.84 | 13.51±9.31 | 0.977 | 1.11±0.82 | 1.1±0.9 | 0.889 |
| rs705702 | GG+AG / AA | 155.14±128.18 | 152.33±140.51 | 0.638 | 13.79±9.86 | 13.31±9.23 | 0.255 | 1.09±0.83 | 1.12±0.91 | 0.357 |
| rs2272046 | AA / AC+CC | 153.68±131.98 | 150.11±144.48 | 0.674 | 13.54±9.11 | 13.29±11.72 | 0.679 | 1.11±0.87 | 1.06±0.84 | 0.338 |
| rs4784165 | GG+GT/ TT | 153.22±127.35 | 153.51±143.49 | 0.962 | 13.53±8.98 | 13.51±10.28 | 0.950 | **1.14±0.92** | **1.05±0.78** | **0.021** |
| rs2059807 | GG+AG / AA | **160.76±151.09** | **145.38±111.92** | **0.009** | 13.77±8.94 | 13.25±10.17 | 0.223 | 1.11±0.87 | 1.09±0.84 | 0.653 |
| rs6022786 | AA+AG / GG | 154.35±141.27 | 151.75±123.54 | 0.667 | 13.53±10.17 | 13.47±8.58 | 0.876 | 1.09±0.82 | 1.12±0.91 | 0.342 |
